# The Scale, Collections, and Biospecimen Distribution of Grade A Tertiary Hospital Biobanks in China: A National Survey

**DOI:** 10.3389/fmed.2020.560600

**Published:** 2021-01-18

**Authors:** Yuanyuan Chen, Chao Sang, Zhouliang Bian, Yinan Zhang, Erpeng Jiang, Xuexun Zhou, Tianlu Chen, Hongming Tang, Congrong Wang

**Affiliations:** ^1^Translational Medical Center for Stem Cell Therapy, Shanghai East Hospital, Tongji University School of Medicine, Shanghai, China; ^2^Translational Medical Center for Stem Cell Therapy & Institute for Regenerative Medicine, Shanghai East Hospital, Tongji University School of Medicine, Shanghai, China; ^3^Center for Translational Medicine, Shanghai Sixth People's Hospital, Shanghai, China; ^4^Ninth People's Hospital, Shanghai Jiao Tong University School of Medicine, Shanghai, China; ^5^The Metabolic Diseases Biobank, Shanghai Key Laboratory of Diabetes, Shanghai Sixth People's Hospital, Shanghai, China; ^6^iFuture Technologies, Shanghai, China; ^7^Department of Endocrinology, Shanghai Fourth People's Hospital Affiliated to Tongji University, Shanghai, China

**Keywords:** biobanks, China, grade A tertiary hospital, survey, biospecimen storage, biospecimen distribution

## Abstract

Chinese clinical biobanks were built rapidly in grade A tertiary hospitals. However, the general information of biorepositories in China remained largely unknown. The aim of this study was to investigate the size, collections, biospecimens distribution and other characteristics of Chinese biobanks in grade A tertiary hospitals. In 2018, we launched a national survey among biobank leaders to provide a comprehensive understanding of Chinese grade A tertiary hospital biobanks. A total of 70 biobank managers or directors completed an online questionnaire to collect information about the biorepositories. Nearly 20% of biobanks stored over one million specimens, while almost one-third of biobanks stored 50–200,000 specimens. In general, plasma and serum were the specimens most commonly stored. For the use of collections, biospecimens were most commonly applied by internal clinical departments. Further analyses revealed that the large-scale biobanks were characterized by earlier establishment, more types of specimens in storage and distribution compared with small-scale biobanks. Moreover, specimens in large-scale biobanks were more commonly used for basic research (62.86% vs. 34.29%, *P* = 0.017) and clinical research (57.14% vs. 28.57%, *P* = 0.016). Large-scale biobanks also had more opportunities to cooperate with domestic research institutes (34.29% vs. 5.71%, *P* = 0.003). Our survey revealed diversity in collections, distribution and utilization of biospecimens among Chinese grade A tertiary hospital biobanks. Although the biobanks had relatively large collections, the underutilization of stored biospecimens and lack of sharing could hamper clinical and biological research.

## Introduction

Human biobanks are repositories of human biological specimens (e.g., blood, tissues, cells, etc.) and associated information (1). These facilities provide valuable resources for all types of biomedical research. Due to the progress in biological research and translational medicine, the field of biobanking has significantly developed. While early biobanks were mostly established to address specific needs of research projects, larger biobanks have now been built to study populations or particular diseases (2). Many large-scale biobanks have been established in developed countries including Denmark, UK, USA, South Korea and Japan (2–4). Some of those repositories are population-based biobanks. For example, UK Biobank is a nationwide biobank funded by the Wellcome Trust medical charity, the UK Department of Health and other institutions. In this project, 500,000 participants aged from 40 to 69 across the country were recruited to provide phenotypic information and biospecimens (blood, saliva and urine) between 2006 and 2010 (3). There are also disease-oriented biobanks supported by academic institutions. AIDS Specimen Bank (ASB) is an example of a university-based biobank built to support HIV research. Until 2012, ASB had received more than 460,000 samples and distributed more than 500,000 aliquots across the world (2). Notably, some private biotechnology companies are also collecting and using human biological materials for commercial reasons. Previous surveys have examined the scale and structure of biobanks in western countries (5–7). In a European survey among biobanks from 23 countries, the majority of the biorepositories were affiliated with academic institutions and only 3% of them were owned by private companies (7). This was confirmed by a U.S. study. In addition, the U.S. survey showed that plasma and serum were stored by most biobanks, while other biological specimens such as hair or toenails were not commonly collected (5).

The demands for high-quality biological samples also increased significantly in China due to the rapid development of medical research (8). In 1994, the first Chinese biobank was established as a national project by the Chinese Academy of Medical Sciences to reserve immortalized cell lines from different ethnic groups (9). In 2003, the Ministry of Science and Technology started the National Infrastructure of Chinese Genetic Resources (NICGR) to integrate genetic resources in China. Since then, clinical biobanks were built rapidly by university hospitals due to the rich resources of biospecimens and personal health data from patients. In 2010, the National Major Scientific and Technological Project for “Significant New Drugs Development project” initiated the biobanking project of Clinical Specimen Repository. The project involves ten grade A tertiary/academic hospitals from Beijing, Tianjin and Shanghai and aims at studying four kinds of major diseases. In the meanwhile, academic hospital biobanks have also been developed in Shanghai and Guangdong (9, 10). Shanghai Clinical Biobank project was launched in 2008 supported by leading grade A tertiary hospitals in Shanghai (9). In Shenzhen, the China National Genebank provides a common platform for hospitals, universities and other research institutions to share information among biobanks (10).

It should be noted that most Chinese clinical biobanks were affiliated with medical institutions. In 2017, a Chinese survey among 42 hospital biobanks showed that most clinical biobanks stored more than 30,000 specimens and were established for <10 years (11). However, the general information of biorepositories in China remained largely unknown. In 2018, we started a national survey among biobank leaders to provide a comprehensive understanding of academic hospital biobanks in China. In the present study, we mainly investigated the size, collections, biospecimens distribution and other characteristics of Chinese biobanks in grade A tertiary hospitals.

## Materials and Methods

### Participants

**Figure 1:**
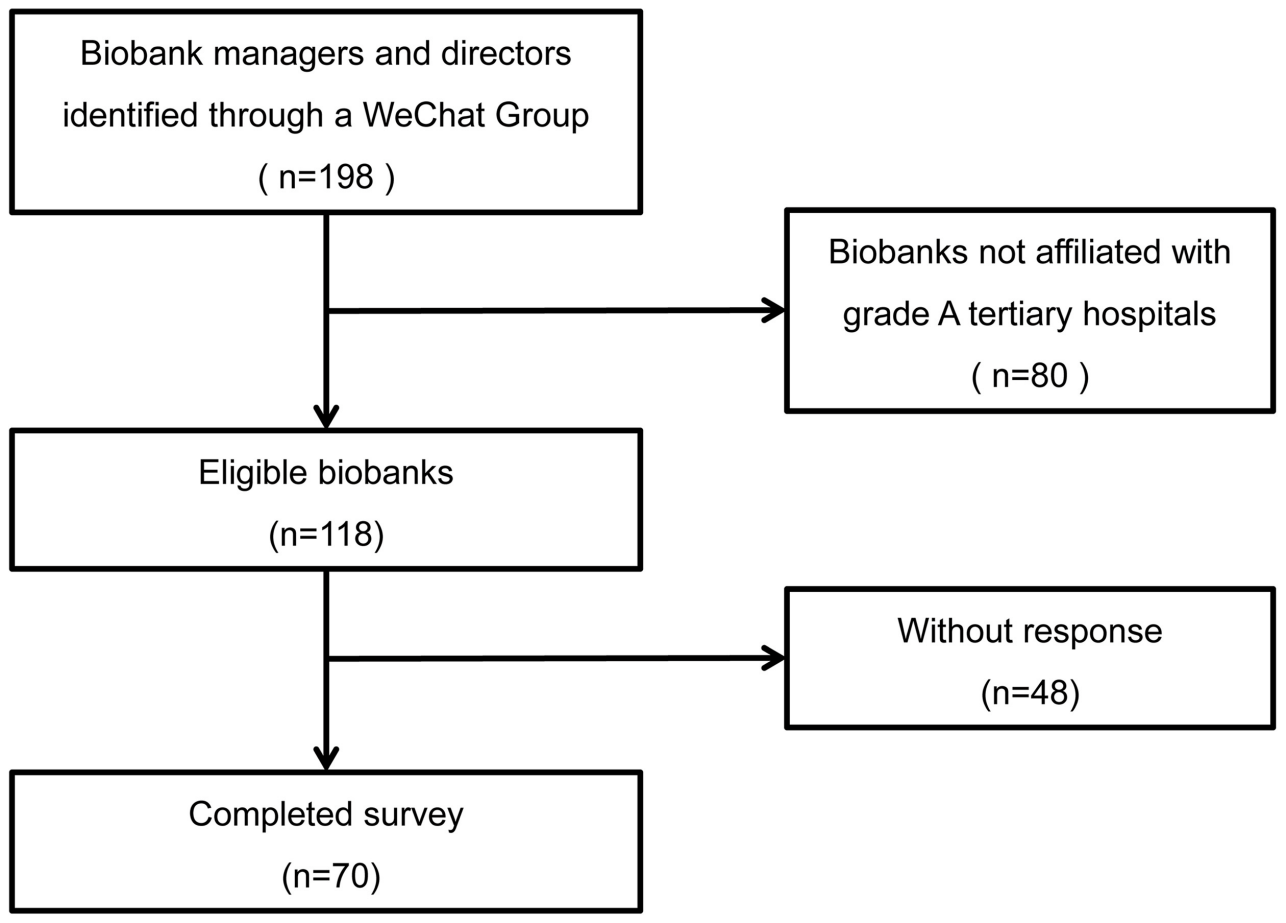
Flow diagram of the study.

A total of 198 biobank managers or directors were identified in a WeChat group of biobank leaders across China (Figure 1). The affiliation of these biobanks included academic hospitals and third-party specimen storage facilities. Biobanks that were not affiliated with grade A tertiary hospitals were excluded from this study (*n* = 80). Of the remaining 118 biobanks, 70 completed the questionnaire. In total, this study achieved a response rate of 59.3%.

### Electronic Survey

In 2018, a survey platform (SO JUMP^TM^) was used to collect information about the biobanks. According to the practical situation of Chinese biobanks, the online questionnaire consists of 59 items including following components: (1) general characteristics (institutions & managers, equipment, services, operating cost & funding support); (2) specimen collection (number and types of samples, location of specimen acquisition, etc.); (3) specimen distribution (number, types and users of samples, access policies, purposes of biospecimen collection, etc.); (4) information management system; (5) biobanking staff (educational background, degree, work experience, income, training, etc.); (6) standard operation procedures and quality control; (7) ethical issues and certifications. This study investigated the relationship between the scale of biobanks and sample collection or distribution of grade A tertiary hospital biobanks in China.

### Statistical Analysis

Statistical analyses were performed using SPSS 21.0 (SPSS Inc., Chicago, IL, USA). Pearson χ^2^ test or continuity chi square test was used to compare the groups with categorical variables. All statistical tests were two-sided. Statistical significance was defined as *P* < 0.05.

## Results

### Biobank Characteristics

A total of 70 biobanks were included in the analyses. Table 1 shows the current storage status among 70 biobanks. Nearly 20% of biobanks (14/70) stored over 1 million specimens, and 18.6% biobanks (13/70) gathered 0.5–1 million specimens, while almost one-third of biobanks (30%, 21/70) stored 50–200,000 specimens. To further investigate the relationship between the scale of the biobanks and their operational features, these biobanks were categorized into two groups, i.e., large-scale biobank (≥200,000 of specimens in storage, *n* = 35) and small-scale biobank (<200,000 of specimens in storage, *n* = 35).

**Table 1:** Number of biobanks in the different categories of sample size.

**Storage sample size**	***n***	**%**
<10,000	5	7.10%
10,000–49,999	9	12.90%
50,000–199,999	21	30.00%
200,000–499,999	8	11.40%
500,000–999,999	13	18.60%
1,000,000–2,000,000	7	10.00%
>2,000,000	7	10.00%

The large-scale biobanks were characterized by earlier establishment, more types of specimens in storage and distribution compared with small-scale biobanks (all *P* < 0.05). Furthermore, large-scale biobanks received and distributed more specimens annually than that of small-scale biobanks (all *P* < 0.05) (Table 2). For information management system, all large-scale biobanks developed biospecimen management system while 20% of small-scale biobanks didn't have such systems. Similarly, a larger proportion of large-scale biobanks developed clinical information system as compared to small-scale biobanks (88.57 vs. 60%, *P* = 0.006). More large-scale biobanks performed quality control on their biological samples than small-scale biobanks (91.43 vs. 68.57%, *P* = 0.017). However, the implementation of standard operation procedure (SOP) was similar between large-scale and small-scale biobanks (Table 2).

**Table 2:** Basic characteristics of the biobanks.

	**Large-scale**		**Small-scale**		***P***
	***n***	**(%)**	***n***	**(%)**	
Year of establishment					
≤ 5 years	12	34.30%	23	65.70%	0.009
>5 years	23	65.70%	12	34.30%	
Types of specimens in storage					
<9	14	40%	23	65.71%	0.031
≥10	21	60%	12	34.29%	
Types of specimens for distribution (2015–2017)					
<6	12	34.29%	23	65.71%	0.009
≥6	23	65.71%	12	34.29%	
Number of new specimens in storage annually (2015–2017)					
≤ 10,000	4	11.40%	14	40.00%	0.006
>10,000	31	88.60%	21	60.00%	
Number of specimens for distributed annually (2015–2017)					
≤ 5,000	14	40.00%	26	74.30%	0.004
>5,000	21	60.00%	9	25.70%	
Biospecimen management system					
Developed	35	100.00%	28	80.00%	0.017
No	0	0.00%	7	20.00%	
Clinical information system					
Developed	31	88.57%	21	60.00%	0.006
No	4	11.43%	14	40.00%	
Biospecimen quality control					
Developed	32	91.43%	24	68.57%	0.017
No	3	8.57%	11	31.43%	
Standard operation procedure (SOP) for biospecimen procurement					
Developed	33	94.30%	29	82.90%	0.260
No	2	5.70%	6	17.10%	
SOP for biospecimen processing					
Developed	29	82.90%	28	80.00%	0.759
No	6	17.10%	7	20.00%	
SOP for biospecimen collection					
Developed	32	91.40%	26	74.30%	0.057
No	3	8.60%	9	25.70%	
SOP for biospecimen distribution					
Developed	30	85.70%	26	74.30%	0.232
No	5	14.30%	9	25.70%	

### Specimens Collection and Storage

**Figure 2:**
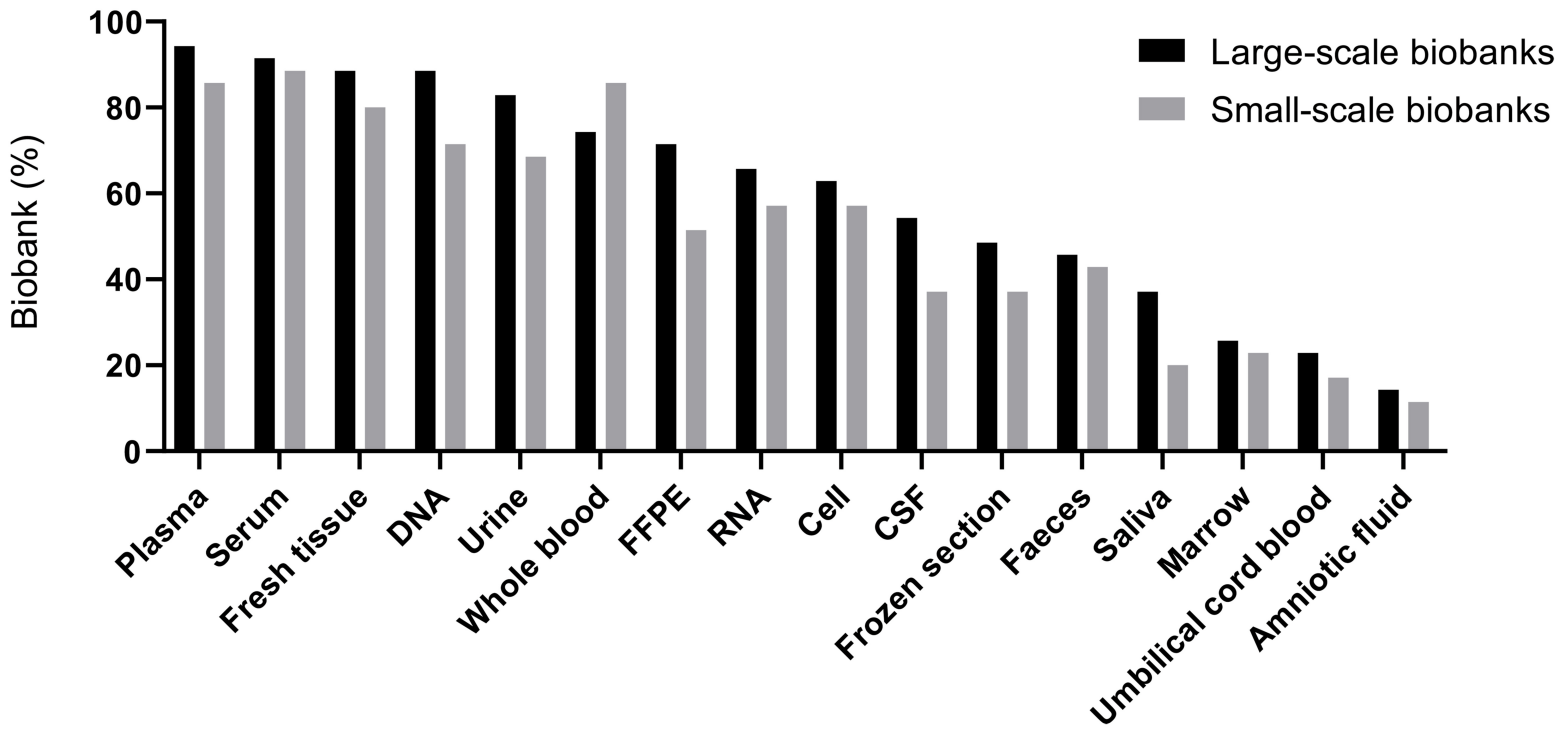
Percentage of biobanks storing different types of specimens. FFPE, formalin-fixed paraffin-embedded tissue; CSF, cerebrospinal fluid.

Detailed analyses showed that the types of specimens in storage were comparable between large- and small-scale biobanks (all *P* > 0.05) (Figure 2). The top 10 specimens stored in these biobanks were quite similar between these two groups as well (Supplementary Table 1). In general, plasma (90%, 63/70) and serum (90%, 63/70) were the specimens most commonly stored. Furthermore, most of the biobanks stored fresh tissue (84.29%, 59/70), DNA (80%, 56/70) and whole blood (80%, 56/70). Saliva (28.57%, 20/70), bone marrow (24.29%, 17/70), umbilical cord blood (20%, 14/70) and amniotic fluid (12.86%, 9/70) were also stored in some biobanks.

We further analyzed the number of types of specimens in storage (Table 3). Most of the biobanks (92.85%, 65/70) stored more than three types of specimens. Over half of biobanks (54.28%, 38/70) stored 8–11 types of specimens.

**Table 3:** Number of types of specimens stored in large- and small-scale biobanks.

**Number of types**	**Large-scale**		**Small-scale**		**All**	
	***n***	**(%)**	***n***	**(%)**	***n***	**(%)**
1	1	2.86%	1	2.86%	2	2.86%
2	0	0.00%	1	2.86%	1	1.43%
3	0	0.00%	2	5.71%	2	2.86%
4	1	2.86%	1	2.86%	2	2.86%
5	0	0.00%	2	5.71%	2	2.86%
6	2	5.71%	2	5.71%	4	5.71%
7	2	5.71%	3	8.57%	5	7.14%
8	4	11.43%	7	20.00%	11	15.71%
9	4	11.43%	4	11.43%	8	11.43%
10	10	28.57%	1	2.86%	11	15.71%
11	2	5.71%	6	17.14%	8	11.43%
12	3	8.57%	1	2.86%	4	5.71%
13	2	5.71%	0	0.00%	2	2.86%
14	1	2.86%	1	2.86%	2	2.86%
15	1	2.86%	2	5.71%	3	4.29%
16	1	2.86%	1	2.86%	2	2.86%
17	1	2.86%	0	0.00%	1	1.43%

### Distribution of Specimens

**Figure 3:**
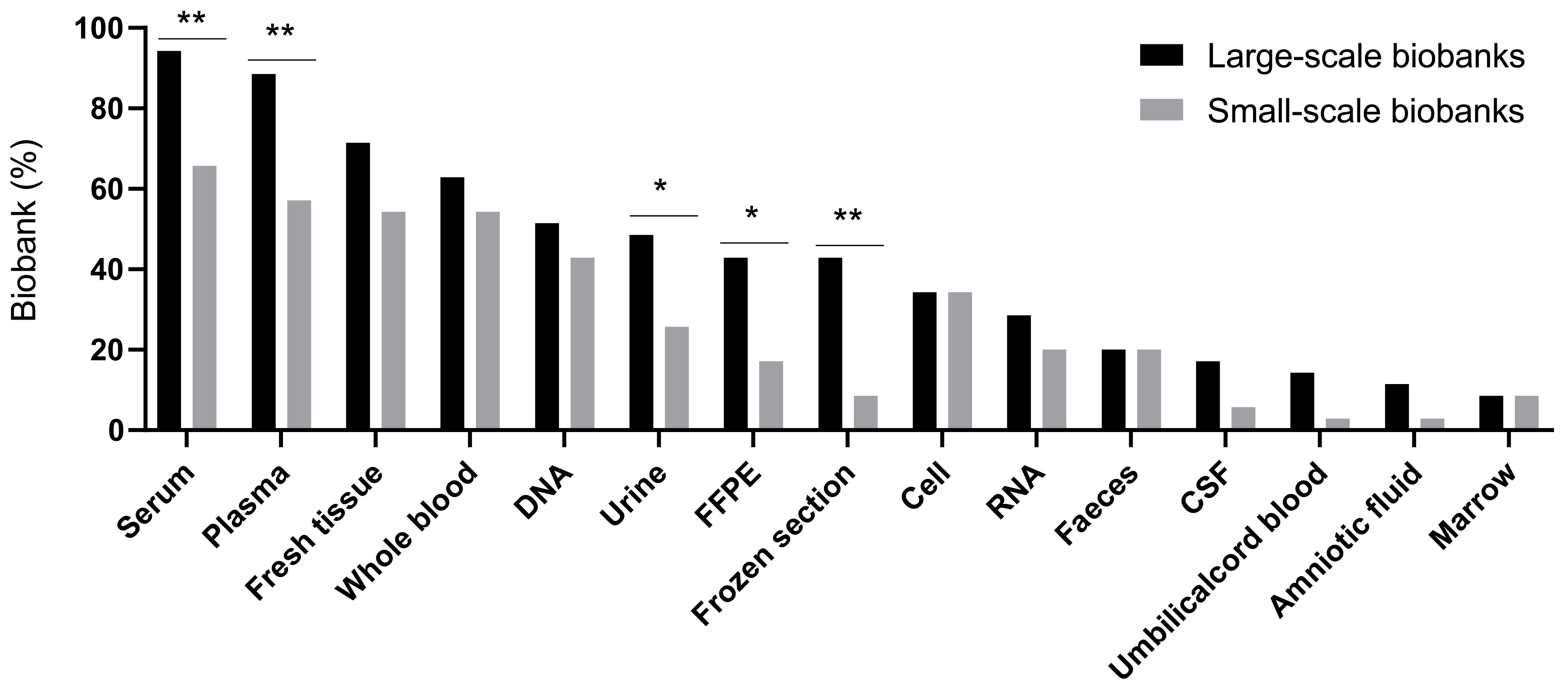
Percentage of biobanks distributing different types of specimens. FFPE, formalin-fixed paraffin-embedded tissue; CSF, cerebrospinal fluid. *P < 0.05, **P < 0.01.

The top 10 specimens distributed in different scale of biobanks in 2015–2017 were also similar (Supplementary Table 1). Nevertheless, serum (94.29 vs. 65.71%, *P* = 0.003), plasma (88.57 vs. 57.14, *P* = 0.003), urine (48.57 vs. 25.71%, *P* = 0.048), formalin-fixed paraffin-embedded tissue (FFPE) (42.86 vs. 17.14%, *P* = 0.019) and frozen section (42.86 vs. 8.57%, *P* = 0.001) were more commonly used by researchers in large-scale biobanks than that in small-scale biobanks as shown in Figure 3. The number of types of specimens for distribution was shown in Table 4. Half of the biobanks (35/70) distributed <6 types of specimens.

**Table 4:** Number of types of specimens distributed by large- and small-scale biobanks (2015–2017).

**Number of types**	**Large-scale**		**Small-scale**		**All**	
	***n***	**(%)**	***n***	**(%)**	***n***	**(%)**
1	2	5.71%	6	17.14%	8	11.43%
2	0	0.00%	4	11.43%	4	5.71%
3	6	17.14%	6	17.14%	12	17.14%
4	1	2.86%	5	14.29%	6	8.57%
5	3	8.57%	2	5.71%	5	7.14%
6	6	17.14%	3	8.57%	9	12.86%
7	3	8.57%	3	8.57%	6	8.57%
8	7	20.00%	3	8.57%	10	14.29%
9	1	2.86%	2	5.71%	3	4.29%
10	2	5.71%	0	0.00%	2	2.86%
11	0	0.00%	1	2.86%	1	1.43%
12	2	5.71%	0	0.00%	2	2.86%
13	2	5.71%	0	0.00%	2	2.86%

We also analyzed the organization and department that used specimens (Table 5). Specimens were most commonly applied by clinical departments (45/70), and the demand for specimens in key clinical departments was also huge (35/70). Compared to small-scale biobanks, specimens in large-scale biobanks seemed more often applied for both basic research (62.86 vs. 34.29%, *P* = 0.017) and clinical research (57.14 vs. 28.57%, *P* = 0.016). Furthermore, large-scale biobanks have more opportunities to cooperate with domestic research institutes (34.29 vs. 5.71%, *P* = 0.003).

**Table 5:** Users and research purposes of biospecimens in large- and small-scale biobanks.

**Users and research purposes**	**Large-scale**		**Small-scale**		***P***
	***n***	**%**	***n***	**%**	
Clinical departments	27	77.14%	18	51.43%	0.025
Key clinical departments	20	57.14%	15	42.86%	0.232
Basic research	22	62.86%	12	34.29%	0.017
Clinical research	20	57.14%	10	28.57%	0.016
Domestic research institutes	17	34.29%	2	5.71%	0.003
International collaboration	5	14.29%	0	0%	0.063
Drug discovery	5	14.29%	2	5.71%	0.426

### Geographical Distribution of Biobanks

We further described the geographical distribution of grade A tertiary hospital biobanks in China. Most large-scale biobanks were built in East China (40.0%) and North China (34.3%). On the other hand, small-scale biobanks were distributed more evenly across different areas of the country (Supplementary Figure 1).

## Discussion

This study is the first national survey to examine the relationship between the scale and collections or biospecimen distribution of grade A tertiary hospital biobanks in China. Our survey revealed diversity in collections, distribution and utilization of biospecimens among the biobanks. This study also showed that the geographic distribution of the grade A tertiary hospital biobanks was unbalanced in China. Most biobanks were built in East and North China which had relatively high levels of economic development. This may reflected that East China and North China had more top research hospitals compared with other areas in China. In addition, several national projects were led by hospitals in East China and North China to promote and activate translational medicine. For example, the project of Significant New Drugs Development was led by Beijing Union Medical College Hospital. While Shanghai Clinical Biobank Project was led by several Shanghai grade A tertiary hospitals.

In the present study, Chinese hospital biobanks varied in size. The number of stored biological samples ranged from <10,000 to over 2,000,000 in different biobanks. Notably, the scale of Chinese academic hospital biobanks was larger than that of western biorepositories. In this study, nearly 40% of biobanks had more than 500,000 biospecimens in storage, while only 8% of the U.S. biobanks stored similar number of samples in 2012. In a European study across 23 countries, 77% of biobanks had <50,000 samples and 25% of them were defined as small-sized biobanks using a cutoff point of 1,000 samples (7). In the meantime, while half of the surveyed biobanks were established within 5 years in this study, 54% of the biorepositories had been built for more than 9 years in the U.S. survey (5). These findings suggested that Chinese hospital biobanks developed rapidly. Moreover, the variability of scale reported in both the U.S. and European studies was consistent with this survey.

For storage and distribution, although almost half of Chinese academic hospital biobanks stored at least 10 types of samples, biobanks affiliated with specialized hospitals such as obstetrics and gynecology hospitals had limited choices and stored relatively few types of specimens. The present study also revealed that large-scale biobanks stored and distributed more types of biological samples. In this study, blood-derived specimens including plasma and serum were the most commonly stored samples among the surveyed biobanks. This finding was consistent with the U.S. survey (5). However, in a European study, DNA was the most commonly collected specimens among 147 biobanks. Although that study only included biobanks preserving or studying genetic information (6). Furthermore, larger proportion of Chinese biobanks stored urine, cord blood and other samples compared with the biobanks in the U.S.

In our study, the types of biospecimens available for distribution were different between small- and large-scale biobanks. Chinese large-scale biobanks distributed significantly more FFPE tissues and frozen tissues than small-scale biobanks. FFPE tissues have been widely collected and used by hospitals for histopathological assessment and diagnosis of various diseases. Interestingly, previous studies suggested that the use of FFPE tissues have increased significantly in cancer research and proteomics studies (12–14). The larger demands for FFPE and frozen tissues in large-scale biobanks may be associated with the increased need for the biobanks to support these fields of research.

In this survey, the usage of biospecimen in Chinese biobanks was diverse, ranging from research to drug discovery. Furthermore, large-scale biorepositories distributed more biospecimens for clinical or basic research compared with small-scale biobanks. However, our study revealed that most biobanks distributed fewer biospecimens than they collected. In line with our findings, a U.S. survey showed that 69% of the 456 biobank managers considered the underutilization of biobank resources as a substantial concern (5). To ensure the long-term sustainability of biobanks, the major goal of them should be improving the utilization of the biological samples instead of simply collecting and storing (15). The failure to effectively use biospecimens may lead to increased expense on storage and hinder the development of biomedical research (16–18).

On the other hand, the insufficient utilization of biospecimens was accompanied by the rapid construction of biobanks in China. The continuous development of biobanks primarily reflect the high demands on high-quality biological samples for research. These biobanks provided important materials for translational research. However, domestic and international collaboration was not active in Chinese grade A tertiary hospital biobanks. The cooperation was even less common in small-scale biobanks. One possible explanation is that the purpose of some biobanks was to provide biospecimens for internal needs of the key departments and research projects within the hospitals. Previous studies also reported that the differences in sample quality, informed consent and access policies could hamper the sharing of samples and data among biobanks (19, 20). Thus, following standard operating procedures for storage, processing and distribution is important for biobanks to provide high-quality biospecimens and promote domestic and international collaboration.

Because most biospecimens in the grade A tertiary hospital biobanks were acquired from the patients, the biobanks were able to collect rare biospecimens in clinical settings. For example, the percentage of biobanks storing cerebrospinal fluid (CSF) in our study was higher than that in the U.S. biobanks (46 vs. 19%) (5). This could be due to the fact that the invasive procedure of obtaining CSF is more commonly performed on patients. There are also several challenges for hospital biobanks to collect and store biospecimens. First, the treatment of diseases may have an impact on the tissues. However, hospitals can provide detailed clinical information related to collected tissues. These data enable researchers to identify the associations of known diseases with biological markers (21). Second, since many samples are collected for diagnostic and other clinical purposes in hospitals, the use of such biospecimens in research should adhere to ethical and scientific guidelines (22).

Our study has limitations. The study population only consisted of grade A tertiary hospital biobanks. However, because of medical resources and were equipped with suitable research facilities, most biobanks were established by grade A tertiary hospitals in China. As the number of biobanks is increasing significantly in China, further investigations were needed to gain a better understanding of Chinese biobanks development.

In conclusion, our study found a significant difference in scale, collections and biospecimen distribution between large-scale and small-scale biobanks. Although surveyed biobanks had relatively large collections, the underutilization of stored biospecimens and lack of sharing could harm the development of clinical and biological researches. Biobank managers should identify the scientific needs of biological samples and recognize the importance of exchanging information to optimize the utilization.

## Data Availability Statement

The raw data supporting the conclusions of this article will be made available by the authors, without undue reservation.

## Ethics Statement

Ethical review and approval was not required for the study on human participants in accordance with the local legislation and institutional requirements. Written informed consent for participation was not required for this study in accordance with the national legislation and the institutional requirements.

## Author Contributions

CW and HT designed the study. YC, CW, and HT contributed to data analysis. YC, CS, ZB, CW, and HT drafted the manuscript. YZ, EJ, XZ, and TC critically revised the manuscript. All the authors have read and approved the final manuscript.

## Conflict of Interest

The authors declare that the research was conducted in the absence of any commercial or financial relationships that could be construed as a potential conflict of interest. The reviewer YL declared a shared affiliation, though no other collaboration, with one of the authors ZB to the handling Editor.

## Supplementary Material

Supplementary Figure 1 Geographical distribution of grade A tertiary hospital biobanks in China.

Supplementary Table 1 Top 10 of specimens in storage and distribution.
