# Supplementary figures and images for: The Scale, Collections, and Biospecimen Distribution of Grade A Tertiary Hospital Biobanks in China: A National Survey
Source: Front Med (Lausanne). 2021 Jan 18;7:560600. doi: 10.3389/fmed.2020.560600 (PMC7848138; doi:10.3389/fmed.2020.560600)

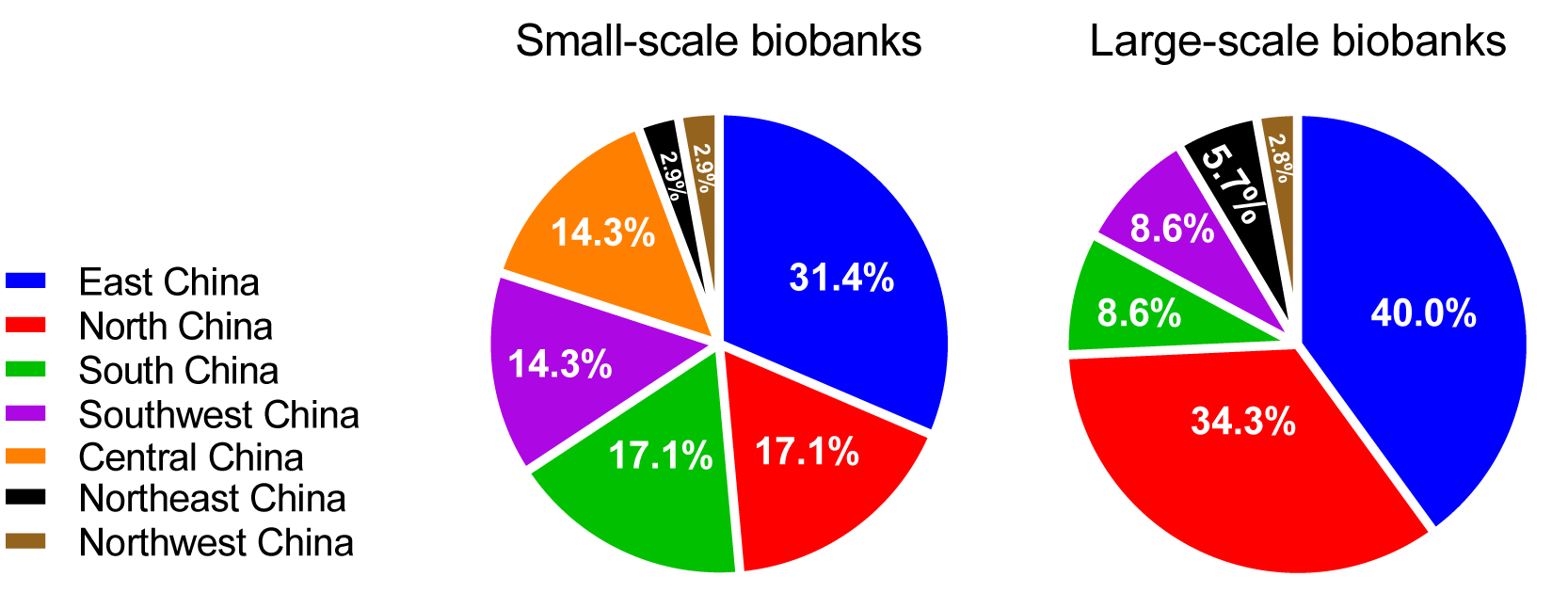

Supplement: Supplementary Figure 1 — Geographical distribution of grade A tertiary hospital biobanks in China. [file Image_1.TIF]
