# Supplementary material for: The Scale, Collections, and Biospecimen Distribution of Grade A Tertiary Hospital Biobanks in China: A National Survey
Source: Front Med (Lausanne). 2021 Jan 18;7:560600. doi: 10.3389/fmed.2020.560600 (PMC7848138; doi:10.3389/fmed.2020.560600)
Supplement: Supplementary Table 1 — Top 10 of specimens in storage and distribution. [file Table_1.docx]

Table S1. Top 10 of specimens in storage and distribution

| Ranking | Storage | | | Distribution（2015-2017） | | |
| --- | --- | --- | --- | --- | --- | --- |
|  | Large-scale | Small-scale | All | Large-scale | Small-scale | All |
| 1 | Plasma | Serum | Plasma | Serum | Serum | Serum |
| 2 | Serum | Whole blood | Serum | Plasma | Plasma | Plasma |
| 3 | Fresh tissue | Plasma | Fresh tissue | Fresh tissue | Fresh tissue | Fresh tissue |
| 4 | DNA | Fresh tissue | Whole blood | Whole blood | Whole blood | Whole blood |
| 5 | Urine | DNA | DNA | DNA | DNA | DNA |
| 6 | Whole blood | Urine | Urine | Urine | Cell | Urine |
| 7 | FFPE | Cell | FFPE | FFPE | Urine | Cell |
| 8 | RNA | RNA | RNA | Frozen section | RNA | FFPE |
| 9 | Cell | FFPE | Cell | Cell | Faeces | Frozen section |
| 10 | CSF | Faeces | CSF | RNA | FFPE | RNA |

FFPE, formalin-fixed paraffin-embedded tissue; CSF, cerebrospinal fluid.
